# Incidental duodenal leiomyoma discovered during robotic SADI: A case report of surgical adaptation and successful outcome

**DOI:** 10.1016/j.ijscr.2025.111648

**Published:** 2025-07-12

**Authors:** Moaz Abulfaraj

**Affiliations:** Department of Surgery, Faculty of Medicine, King Abdulaziz University, Jeddah, Saudi Arabia

**Keywords:** Duodenal leiomyoma, Robotic SADI, Robotic mini gastric bypass, Bariatric surgery, Incidental finding, Case report

## Abstract

**Introduction and importance:**

Duodenal leiomyomas are rare, often asymptomatic benign tumors, typically discovered incidentally during surgery. This case highlights a unique leiomyoma found during robotic Single Anastomosis Duodeno-Ileal bypass (SADI), emphasizing the importance of intraoperative adaptability in bariatric surgery.

**Case presentation:**

A 56-year-old male with a BMI of 56 kg/m^2^ underwent robotic SADI. Despite unremarkable preoperative endoscopy, sutures cut through a thickened duodenal wall during duodeno-ileostomy, revealing a leiomyoma on frozen section biopsy. The duodenum and distal antrum were resected, and the procedure was converted to robotic mini gastric bypass (MGB).

**Clinical discussion:**

This rare finding underscores the challenge of detecting submucosal lesions preoperatively and the need for rapid intraoperative decision-making. Conversion to MGB achieved 90 % excess weight loss at 1-year follow-up, with no malnutrition or complications, demonstrating effective adaptation.

**Conclusion:**

Surgeons performing robotic bariatric procedures must be prepared to manage unexpected pathology through flexible surgical strategies, ensuring favorable outcomes.

## Highlights

• Reports a rare duodenal leiomyoma found incidentally during robotic SADI, a modern bariatric procedure.
• Demonstrates successful intraoperative adaptation by converting to robotic mini gastric bypass (MGB).
• Achieved 90 % excess weight loss and diabetes remission at 1-year follow-up, with no complications.
• Highlights the challenge of detecting submucosal lesions preoperatively, emphasizing surgical vigilance.
• Validated by histopathology and frozen section biopsy, confirming benign leiomyoma.

## 1 Introduction

Duodenal leiomyomas are exceptionally rare benign neoplasms originating from the smooth muscle layers of the gastrointestinal tract, comprising <1 % of all GI leiomyomas [1,2]. Historically, these tumors have been documented sporadically, often discovered incidentally during autopsy, imaging, or surgical exploration for unrelated conditions [1,3]. Their asymptomatic nature and submucosal location frequently evade preoperative detection, posing diagnostic and management challenges intraoperatively [4]. In the context of bariatric surgery, where anatomical alterations are routine, such unexpected findings can significantly influence procedural planning and outcomes.

Single Anastomosis Duodeno-Ileal bypass (SADI), introduced by Sánchez-Pernaute et al. in 2007, is an evolving bariatric technique designed for severe obesity [5]. It combines sleeve gastrectomy with a duodeno-ileal anastomosis, preserving the pylorus to mitigate nutritional deficiencies compared to traditional malabsorptive procedures like biliopancreatic diversion [6]. The adoption of robotic platforms enhances precision in SADI, offering improved visualization and dexterity, particularly for complex anastomoses [7]. SADI's efficacy in achieving substantial weight loss has led to its increasing use worldwide [8]. However, the procedure assumes a structurally intact duodenum, and intraoperative anomalies—such as tumors—may necessitate immediate adaptation. Robotic mini gastric bypass (MGB), an alternative bariatric approach, offers a simpler single-anastomosis solution when anatomical constraints arise [9].

We present a rare case of an incidental duodenal leiomyoma discovered during robotic SADI in a 56-year-old male with morbid obesity. The finding prompted resection and conversion to robotic MGB, resulting in a successful 1-year outcome. This report highlights the critical role of intraoperative decision-making and adaptability in managing unexpected pathology during robotic bariatric surgery, contributing to the sparse literature on duodenal leiomyomas in this setting.

## 2 Case presentation

A 56-year-old male with morbid obesity (BMI 56 kg/m^2^, weight 168 kg, height 173 cm) presented for bariatric surgery. His comorbidities included hypertension and type 2 diabetes mellitus, controlled with lisinopril and metformin, respectively. Preoperative workup, including laboratory tests (hemoglobin 13.5 g/dL, albumin 4.0 g/dL) and upper GI endoscopy, revealed no abnormalities. Robotic SADI was selected for its balance of weight loss efficacy and reduced malabsorption, leveraging robotic precision as a tool.

The procedure was performed using the da Vinci Xi robotic system. After docking the robot, sleeve gastrectomy commenced with division of the greater omentum using a robotic harmonic scalpel. The stomach was resected along the lesser curvature with a 40-French bougie as a guide, employing a SureForm stapler with a blue load, controlled robotically, from the antrum to the angle of His, preserving the pylorus. The duodenum was mobilized 2 cm distal to the pylorus using robotic sharp dissection, and the ileum was measured 250 cm proximal to the ileocecal valve for anastomosis.

**Fig. 1:**
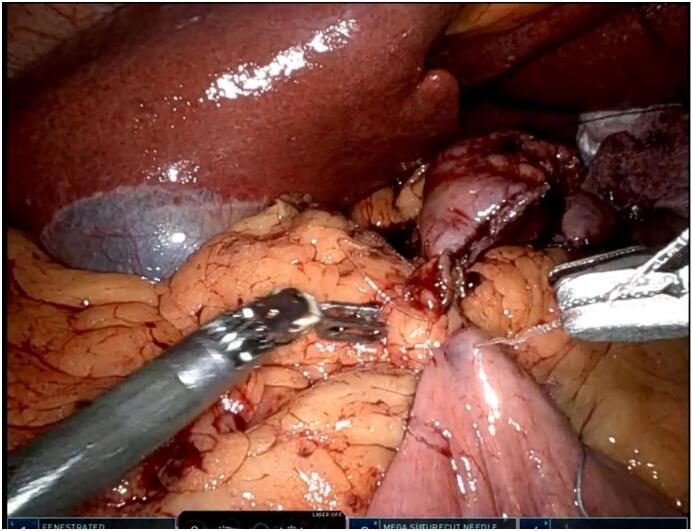
Intraoperative image of sutures cutting through duodenal wall. Caption: Robotic intraoperative view showing 2-0 V-Loc suture cutting through the thickened duodenal wall during the first posterior layer of the duodeno-ileostomy.

For the duodeno-ileostomy, the duodenum was transected with a robotic SureForm stapler using a white load. An end-to-side anastomosis was initiated using a 2-0 V-Loc suture to construct the first posterior layer. During this step, the suture repeatedly cut through the duodenal wall under minimal tension, exposing an abnormal thickness not evident preoperatively (Fig. 1). Inspection with robotic 3D visualization revealed a 3 × 2 cm firm, submucosal mass in the first part of the duodenum (D1). A biopsy was taken and sent for frozen section analysis, which identified a leiomyoma (spindle cells, low mitotic rate, no atypia). Due to the compromised duodenal wall, resection was undertaken.

**Fig. 2:**
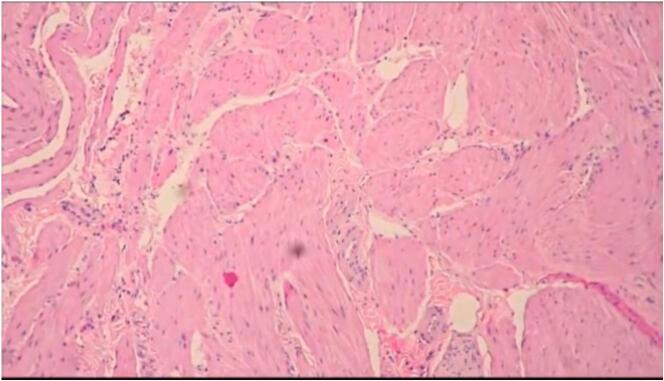
Histology of duodenal leiomyoma. Caption: Microscopic view (H&E stain, 10×) showing spindle-shaped smooth muscle cells with low mitotic activity, consistent with benign leiomyoma.

Resection of the affected segment (D1 and proximal D2, ~5 cm) and a 1 cm portion of the distal gastric antrum was performed to ensure clear margins. The specimen was sent for repeat frozen section, confirming negative margins. With the duodenum resected, completing robotic SADI was unfeasible. Conversion to a robotic mini gastric bypass (MGB) was carried out: a 200 cm biliopancreatic limb was measured from the ligament of Treitz, and a gastrojejunostomy was created between the gastric pouch and jejunum using a robotic SureForm stapler. An intraoperative methylene blue test confirmed anastomotic integrity. Operative time was 240 min, with 100 mL blood loss. The procedure was performed by a bariatric surgeon experienced in robotic SADI and MGB. Postoperative recovery was uneventful; liquids were started the next morning, and discharge occurred on postoperative day 1. Histopathology confirmed a benign duodenal leiomyoma (3.5 × 2.2 × 1.8 cm, spindle cell pattern, Ki-67 < 1 %) (Fig. 2).

Timeline: The patient underwent elective robotic SADI on 15/03/2024. Preoperative workup (endoscopy, labs) was unremarkable. Intraoperative discovery of a leiomyoma led to resection and conversion to MGB on the same day. The patient was discharged on 16/03/2024.

Patient Perspective: At the 1-year follow-up (March 2025), the patient expressed satisfaction with the surgical outcome, reporting improved quality of life due to significant weight loss and resolution of diabetes, with no adverse effects from the procedure. The patient achieved 90 % EWL (weight 90 kg, BMI 30 kg/m^2^), with no malnutrition (albumin 3.9 g/dL, hemoglobin 13.2 g/dL) or dumping syndrome. Bowel movements averaged 2–3 daily, well-tolerated, and diabetes was in remission (HbA1c 5.8 % off medication). This case report has been reported in line with the SCARE 2025 Guidelines [10].

## 3 Discussion

Duodenal leiomyomas are among the rarest GI tumors, with fewer than 50 cases reported since the mid-20th century [1,3]. A 1977 review by Serour et al. documented their incidental nature, often found at autopsy, while a 1981 case by Freund et al. described a symptomatic duodenal leiomyoma resected during laparotomy for abdominal pain [1,3]. In this case, the tumor was asymptomatic and undetected by preoperative endoscopy, likely due to its submucosal location, a known limitation of standard endoscopic techniques [4,11]. This mirrors rare reports of unexpected GI tumors in bariatric surgery, such as a gastric leiomyoma during Roux-en-Y gastric bypass (RYGB) reported by Almazeedi et al. in 2013, where resection and RYGB completion were feasible [12]. Some surgeons routinely perform preoperative esophagogastroduodenoscopy (EGD) in bariatric patients to identify mucosal abnormalities like ulcers, gastritis, esophagitis, or hiatal hernia that may alter surgical plans [4]. However, its sensitivity for submucosal lesions, such as the leiomyoma in this case, is limited, as these tumors often lie beneath the mucosa and may not disrupt the surface visible on EGD [10]. This case illustrates how preoperative EGD, while useful for detecting overt pathology, can miss critical submucosal findings.

Robotic SADI leverages the da Vinci system as a tool for enhanced precision through 3D visualization and articulated instruments, yet its reliance on a structurally sound duodenum was challenged here [5,7]. The suture cut through during the first posterior layer, captured intraoperatively (Fig. 1), likely resulted from the leiomyoma's disruption of wall tensile strength, a phenomenon not previously reported in SADI literature. Conversion to robotic MGB, established by Lee et al. in 2005, was a pragmatic solution, utilizing a single gastrojejunostomy to bypass the resected duodenum [9]. The patient's 90 % EWL at 1 year aligns with reported outcomes for both SADI (80–85 %) and MGB (70–90 %) [6,9], reflecting his high initial BMI and metabolic response.

Intraoperative frozen section analysis was pivotal in distinguishing benign leiomyoma from malignancy (e.g., leiomyosarcoma), adhering to guidelines for unexpected masses [13]. This case highlights the rarity of duodenal pathology complicating robotic SADI and the adaptability required in bariatric surgery. A limitation of this report is its single-case nature, precluding broader conclusions about the prevalence of such findings. Future studies could investigate the frequency of incidental tumors in robotic bariatric procedures to inform preoperative strategies and optimize intraoperative management.

## 4 Conclusion

This case represents a rare instance of an incidental duodenal leiomyoma complicating robotic SADI, necessitating resection and conversion to robotic MGB. The successful 1-year outcome—90 % EWL, diabetes remission, and no nutritional deficits—demonstrates the efficacy of intraoperative adaptability in managing unexpected pathology with robotic assistance as a tool. Surgeons performing robotic SADI, or similar procedures should remain vigilant for gastrointestinal anomalies, prepared to modify techniques and utilize rapid pathology assessment. This report adds to the limited literature on duodenal leiomyomas in bariatric contexts, advocating for heightened awareness, flexibility in surgical planning, and further investigation into the frequency of such findings to optimize patient outcomes.

## Author contribution

**Moaz Abulfaraj**: Conceptualization, surgical procedure, data collection, data interpretation, writing the original draft, reviewing and editing the manuscript, and final approval for submission.

## Consent

Written informed consent was obtained from the patient for publication of this case report and accompanying images. A copy of the written consent is available for review by the Editor-in-Chief of this journal on request.

## Ethical approval

Ethical approval was not required for this case report per the institutional policy, as it involves a single patient with no experimental intervention. All procedures adhered to the ethical standards outlined in the Declaration of Helsinki.

## Additional information

The case has not been presented at a conference or submitted to another journal.

## Guarantor

Moaz Abulfaraj.

## Research registration number

Not applicable.

## Declaration of Generative AI and AI-assisted technologies in the writing process

An AI-based proofreading tool (Grammarly, version 2025, cloud-based API, accessed March–June 2025) was used to enhance language clarity and readability during manuscript drafting and revisions. No generative AI was employed. The tool was used solely for grammatical corrections and stylistic suggestions, affecting the writing stage. All outputs were reviewed and edited by the author, who takes full responsibility for the content's integrity. No patient data or clinical images were input into the AI tool, ensuring compliance with GDPR and HIPAA. The author, a bariatric surgeon, verified all AI suggestions for clinical accuracy and discarded inappropriate edits. No algorithmic bias was relevant, as the tool was used for language only, with no financial ties to the vendor. Reproducibility details are not applicable, as the tool provided automated suggestions without specific prompts.

## Funding

No funding was received for this case report.

## Conflict of interest statement

The author declares no conflicts of interest.
